# Synthesis and Biological Evaluation of 7-Deoxy-Epothilone Analogues

**DOI:** 10.3390/ijms18030648

**Published:** 2017-03-17

**Authors:** Laura M. Woods, Joseph W. Arico, Jeffrey D. Frein, Dan L. Sackett, Richard E. Taylor

**Affiliations:** 1 Department of Chemistry and Biochemistry, the Harper Cancer Research Institute, and the Warren Family Research Center for Drug Discovery & Development, University of Notre Dame, Notre Dame, IN 46556, USA; lwoods4@nd.edu (L.M.W.); jarico81@gmail.com (J.W.A.); jdfrein@gmail.com (J.D.F.); 2 Eunice Kennedy Shriver National Institute of Child Health and Human Development, National Institutes of Health, Bethesda, MD 20892, USA; sackettd@mail.nih.gov

**Keywords:** epothilone, conformation, binding site, cancer

## Abstract

The synthesis of two deoxygenated analogues of potent epothilones is reported in an effort to analyze the relative importance of molecular conformation and ligand–target interactions to biological activity. 7-deoxy-epothilone D and 7-deoxy-(*S*)-14-methoxy-epothilone D were prepared through total synthesis and shown to maintain the conformational preferences of their biologically active parent congeners through computer modeling and nuclear magnetic resonance (NMR) studies. The significant decrease in observed potency for each compound suggests that a hydrogen bond between the C7-hydroxyl group and the tubulin binding site plays a critical role in the energetics of binding in the epothilone class of polyketides.

## 1. Introduction

Modulation of microtubule dynamics by natural products through either inhibiting polymerization (e.g., vincristine, vinblastine, colchicine) or by stabilizing the polymeric form (e.g., paclitaxel (Taxol^®^), docetaxel (Taxotere^®^), and the epothilones (Ixempra^®^)) leads to mitotic arrest and apoptosis and is the basis of many clinically relevant therapeutic agents [1,2,3,4,5]. While the microtubule-depolymerizing vinca alkaloids have been used clinically for more than fifty years, the more recent advent of tubulin-stabilizing agents has led to notable success in developing new generations of anticancer agents for solid tumors. For example, Taxol^®^ is a Food and Drug Administration (FDA)-approved treatment option alone or in combination with other chemotherapeutic agents for ovarian carcinoma, node-positive breast cancer, non-small cell lung cancer, and Kaposi’s sarcoma [6].

**Figure 1:**
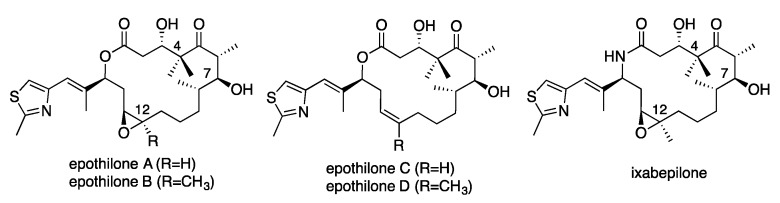
Naturally occurring epothilones A, B, C, and D and the semi-synthetic analogue ixabepilone.

The epothilones are polyketide natural products first isolated in 1987 by Reichenbach and Höfle from the myxobacterium strain *Sorangium cellulosum* (Figure 1) [7,8]. Epothilones have been demonstrated by Bollag et al. [9] to interact with β-tubulin at the paclitaxel binding site, inducing tubulin assembly and stabilization of the polymeric form [10,11]. Compared to Taxol^®^, epothilones show reduced peripheral neuropathy, resistance to P-glycoprotein efflux pumps, and increased water solubility in vitro [9]. These and other advantages associated with the epothilones have spurred enormous, multi-disciplinary efforts to evaluate and enhance their therapeutic potential [12,13]. Ixabepilone (Ixempra^®^), a semi-synthetic epothilone B analogue, in 2007, became the first epothilone to be granted FDA approval, for metastatic breast cancer and locally advanced, taxane-resistant breast cancer as a monotherapy or in combination with capecitabine (Figure 1) [14].

A structure–activity profile can be generated from an iterative process of chemical modifications and biological testing to identify critical structural features necessary for a compounds biological activity. In the case of the epothilones, results from these studies show the importance of the macrolide ring and specifically the stereochemistry of the C3–C8 polypropionate region. Also, in epothilone A and B, the 12*R*, 13*S* configuration of the epoxide is essential for activity [15]. Furthermore, the olefin connecting the side chain at C16–C17 is required, indicating the importance of the spatial relationship between the side chain and the thiazole ring [16,17]. Our lab has been interested in the role of the functionality that adorns a polyketide in controlling the overall conformation [18]. These studies led to defining a bioactive conformational profile for the epothilones based on computational analysis and nuclear magnetic resonance (NMR) studies in multiple solvents [19,20,21,22,23]. Our efforts uncovered two areas of conformational flexibility resulting in a relatively small group of conformational families [19], one of which is similar to the conformation observed in the solid state [7]. The C4–C8 polypropionate region primarily exists in two conformers controlled by a combination of *syn-*pentane interactions and hydrogen bonding. We previously synthesized (*S*)-10-methyl-epothilone C adding a new *syn*-pentane interaction between C10 and C8 methyl groups and effectively eliminating one major conformation [22]. This analogue showed a complete loss of cytotoxicity, compared to the parent compounds, suggesting that its conformational profile was not likely related to the bound conformation. Further support for this conclusion was the potent activity of (*E*)-9,10-dehydro-epothilone B which stabilizes the solid-state conformation in this region through the minimization of A_1,3_-strain [20]. A second flexible region was observed in the C11–C15 region of the macrolide providing two local conformers. Diasteroisomeric 14-substituted epothilone analogues would control the local conformation again through A_1,3_-strain [21]. (*S*)-14-methyl-epothilone D and (*R*)-14-methyl-epothilone B stabilized the conformation observed in the solid-state, and retained activity while (*R*)-14-methyl-epothilone D lost all biological activity. Our conclusion that the bioactive conformation was closely related to that observed in the solid-state was further supported by synthesis and biological evaluation of (*S*)-14-methoxy-epothilone D, a potential biosynthetically derived analogue [23]. The combined results highlighted the critical importance of retaining the bound conformation in simple analogues of polyketides [18].

Following on from these valuable insights into the role of functionality in conformation, we became interested in further examining the pharmacophore and the potential role of the C7-hydroxyl group. The C7-hydroxyl was previously proposed to exhibit a strong hydrogen bonding interaction within the paclitaxel binding site at T274, based on electron crystallography (EC) of zinc stabilized tubulin sheets [24,25,26]. Site mutation (Thr274Ile) resulted in epothilone resistance, indicating a change in the structure of the binding site or an intermolecular hydrogen-bonding interaction with epothilone [24,25]. This EC-model deviated significantly from NMR-based studies and, therefore, the validity of their conclusions has been debated [19,20,21,22,27,28,29,30].

Though many structure–activity relationships have been examined with the epothilone class of natural products, only two studies have probed the C7 position. Nicolaou reported the inversion of both the C7-hydroxyl and C6-methyl concurrently to give the 6*S*,7*R* stereoisomer. Biological experiments with this compound failed to induce tubulin polymerization, but this is likely a conformational effect [15]. Höfle used semi-synthesis to generate the C7-ketone, and this analogue did not lose complete activity [31]. However, manipulation of the C7 site alone, to probe the importance of either intramolecular or intermolecular hydrogen bonding has not been examined.

As described earlier, we have previously shown that in solution the C1–C8 polypropionate region prefers to exist in at least two conformational families controlled by *syn*-pentane interactions and intramolecular hydrogen bonds [19]. NMR studies suggested an increase in population of the biologically inactive conformation in non-polar solvents due to an intramolecular hydrogen bond between the C7-hydroxyl and the C5-carbonyl. Therefore, in contrast to the earlier reported necessity of the C7-hydroxyl for intermolecular interactions within the binding site, we hypothesized that removal of the C7-hydroxyl might enhance biological activity by increasing the population of the bioactive conformer. To determine the role of the C7-hydroxyl functionality, we synthesized 7-deoxy-epothilone D and a structurally related analogue, 7-deoxy-(*S*)-14-methoxy-epothilone D, two compounds which should maintain a significant preference for the bound conformation. The subsequent biological activity of these compounds would then provide insight into the relative importance of conformational preference or binding site interactions related to the C7-hydroxyl.

## 2. Results and Discussion

**Scheme 1:**
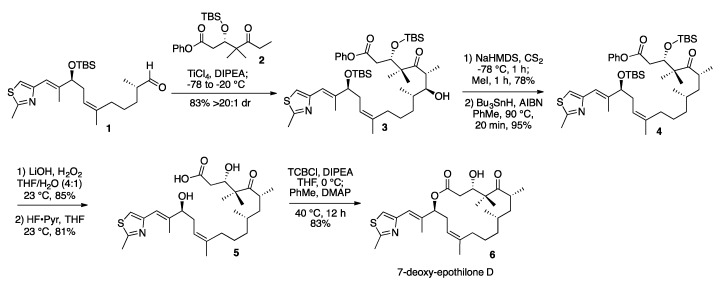
Synthesis of 7-deoxy-epothilone D. THF: tetrohydrofuran; Pyr: pyridine; PhMe: toluene; NaHMDS: sodium hexamethyldisilazide; TBS: *tert*-butyldimethylsilyl; DIPEA: diisopropylethylamine; AIBN: azobisisobutyronitrile; DMAP: 4-dimethylamino-pyridine; TCBCL: trichlorobenzoylchloride.

As shown in Scheme 1, the synthesis of 7-deoxy-epothilone D commenced with known aldehyde **1** [32,33,34,35], which underwent a highly selective aldol reaction (>20:1) with **2** in 83% yield, as previously reported [36]. This was followed by introduction of the methyl xanthate, effected by deprotonation of the C7-hydroxyl with sodium hexamethyldisilazide (NaHMDS) in the presence of CS_2_, and subsequent quenching with methyl iodide. Radical-mediated reduction with tributyltin hydride gave the 7-deoxy intermediate **4** in high yield (95%) [37]. Saponification of the ester with lithium hydroxide followed by global deprotection with HF·pyridine gave diol **5**. Finally, Yamaguchi macrolactonization afforded the first analogue, 7-deoxy-epothilone D [38].

**Scheme 2:**
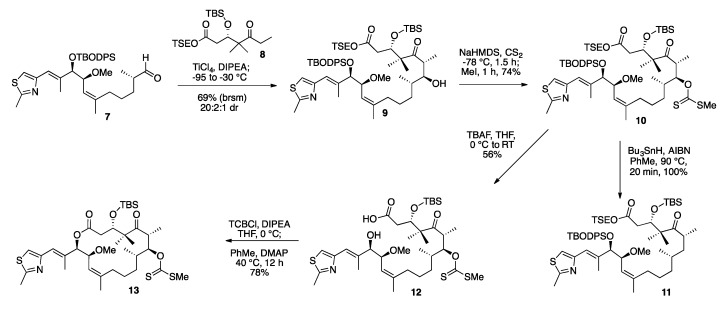
Synthesis of 7-deoxy-(*S*-14-methoxy-epothilone D macrocyclic intermediate. TBODPS: *tert*-butydiphenoxysilyl; TSEO: 2-(trimethylsilyl)ethoxy; TBAF: tetrabutylammonium fluoride.

The 7-deoxy-(*S*)-14-methoxy-epothilone D analogue was prepared using a modified version of our previously published route to (*S*)-14-methoxy-epothilone D [23]. A TiCl_4_-mediated aldol to append the top fragment was envisioned (see first step of Scheme 1) but featuring aldehyde **7** [23] (bearing the (*S*)-14-methoxy group) and TSE (2-trimethylsilylethoxy) ester (**8**). The use of a TSE protecting group instead of the phenyl ester present in **2** would advantageously allow simultaneous unmasking of the C15-hydroxyl group and the carboxylic acid necessary for the macrolactonization, thus saving an additional deprotection step from the previous synthesis. As shown in Scheme 2, aldehyde **7** and TSE ester **8** underwent a highly selective (20:2:1) aldol reaction to afford **9**. Methyl xanthate formation (**10**) proceeded smoothly in 74% yield. While deoxygenation progressed rapidly in quantitative yield, the desired product **11** was obtained as a 9:1 inseparable mixture of products. The minor product was not readily identified and could not be separated by typical chromatographic techniques. The ^1^H NMR spectra of the two products nearly overlapped, suggesting that the two products were diastereomers or closely related.

**Scheme 3:**
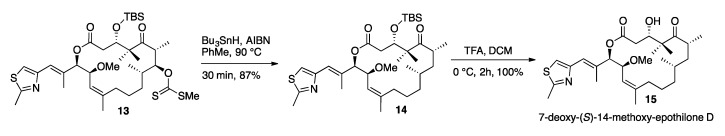
Synthesis of 7-deoxy-(*S*)-14-methoxy-epothilone D. TFA: trifluoroacetic acid; DCM: dicholoromethane.

In an effort to avoid carrying a mixture of products through multiple steps, it was decided to perform the C7-deoxygenation at a later stage in the route. Accordingly, the *tert*-butydiphenoxysilyl (TBODPS) and 2-(trimethylsilyl)ethoxy (TSE) protecting groups were removed with tetrabutylammonium fluoride (TBAF) in 56% yield (**12**). Macrolactonization proceeded smoothly in 78% yield to afford methyl xanthate macrocycle **13**. As shown in Scheme 3, the xanthate was then removed using a large excess (15 equivalents) of Bu_3_SnH to provide the desired protected deoxymacrolactone, **14** in high yield (87%). A minor diastereomer was observed similar to that described above, but the major product could be purified by preparative thin-layer chromatography (TLC). Deprotection of the *tert*-butyldimethylsilyl (TBS) group with excess trifluoroacetic acid (TFA) produced the second desired target, 7-deoxy-(*S*)-14-methoxy-epothilone D, in quantitative yield.

After completion of the syntheses, the novel epothilone analogues were tested in A2780 (1A9) human ovarian carcinoma cell lines and compared to Taxol^®^ and natural epothilones. The inhibitory activities are shown as GI_50_ values in Table 1. Comparing the activity of epothilone D to the 7-deoxy-epothilone D, removal of the hydroxy group caused more than a 100-fold loss in activity (7.5 nM vs. 0.9 μM). Deoxygenation of C7 within the 14-methoxy analogue also showed a significant drop in potency (29 nM vs. 2.2 μM). Interestingly, conformational analysis of the deoxy-analogues suggested significant similarities to their potently cytotoxic parent compounds. 7-deoxy-epothilone D maintained a key ROESY interaction between H3 and H6. Furthermore, the coupling observed between H6 and the two protons of H7 is indicative of the conformational averaging previously observed with epothilone D [19]. The ^1^H NMR coupling constants of H6 consist of 7.2 Hz to the C6-methyl and two mid-range couplings of 4.2 and 9 Hz to the diastereomeric C7 protons. Comparable couplings were observed for H6 in 7-deoxy-(*S*)-14-methoxy-epothilone D. A methyl coupling of 6.6 Hz and two mid-range couplings of 4.8 and 9.6 Hz, corresponding to interaction with H7a/b. In contrast to natural epothilone D, the coupling between H6 and H7 is 2.4 Hz [39]. The coupling constants of the analogues are both higher than the natural compound, suggesting a greater population of the major conformer.

Despite an enhanced conformational preference for the biologically active conformation, both new compounds exhibit a significantly lower potency, which provides further support for the importance of an intermolecular interaction between the C7-hydroxyl and the tubulin binding site. While early studies proposed a hydrogen bond with T276, more recent high resolution (2.3 Å) X-ray crystal structure and independent NMR studies of epothilone A bound to tubulin dimers both revealed that the C7-hydroxyl interacts with D226, and the T276 interaction is with N20 [40,41].

**Figure 2:**
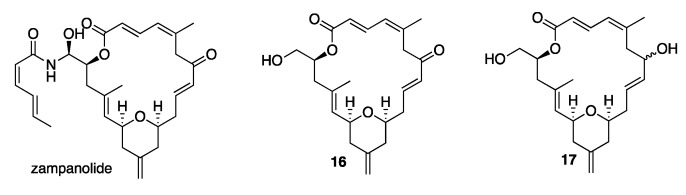
Zampanolide and analogues of the structurally related dactylolide natural product.

The critical importance of this single hydrogen bond may be analogous to the interaction of zampanolide within the identical binding site on tubulin [40,41]. A high-resolution X-ray crystal structure showed that interactions between zampanolide and tubulin are limited but include a critical covalent interaction. The α,β-unsaturated enone moiety alkylates the imidazole of H229 and removal of this functionality in analogue (**17**) of the structurally related compound dactylolide abolishes all activity (IC_50_ **17** = 24,000 nM vs. **16** = 320 nM in PC-3 cells, Figure 2) [42,43]. While hydrogen bonds are less energetically stable than covalent bonds, hydrogen bonds can vary significantly in intrinsic strength, enough to rationalize the loss of cytotoxicity in the analogues presented here. Overall, the combined results of our studies show that while conformation is of critical importance in analogue design strategies, certain individual, energetically favorable intermolecular interactions cannot be disregarded [44,45].

## 3. Materials and Methods

### 3.1. General Information

Unless otherwise noted, all materials were used as received from a commercial supplier without further purification. All anhydrous reactions were performed using oven-dried or flame-dried glassware under an atmosphere of nitrogen or argon; unless otherwise indicated, all reactions were performed under anhydrous conditions. Tetrahydrofuran (THF), diethyl ether (Et_2_O), dichloromethane (CH_2_Cl_2_), and toluene were filtered through activated alumina under nitrogen prior to use. Pentane and triethylamine (NEt_3_) were dried over LiAlH_4_ and CaH_2_, respectively, and distilled prior to use. Molecular sieves (4 Å) were oven-dried overnight and cooled under high vacuum prior to use. Anhydrous *N*,*N*-dimethylformamide (DMF) was purchased from Acros, Geel, Belgium (AcroSeal^®^ bottle, over 4 Å molecular sieves). Thionyl chloride (SOCl_2_) was distilled prior to use. All reactions were monitored by either E. Merck (Kenilworth, NJ, USA) analytical thin layer chromatography (TLC) plates (silica gel 60 GF, glass back) or Whatman (Maidstone, UK) ultraviolet (UV) active aluminum backed TLC plates (silica gel 250 μm) and analyzed with 254 nm UV light and/or *p*-anisaldehyde/sulfuric acid or potassium permanganate treatment. Silica gel for column chromatography was purchased from E. Merck (Silica Gel 60, 230–400 mesh). Biotage chromatography was performed using Flash 40+M, 25+M, 25+S, or 12+M KP-Sil™ Silica Cartridges (32–63 μm, 60 Å, nominally 500 m^2^/g silica)—Biotage GmbH, Düsseldorf, Germany. All ^1^H and ^13^C NMR spectra were obtained either on a Varian INOVA 500 spectrometer (operating at 499.864 MHz for ^1^H and 125.690 MHz for ^13^C) or on a Varian INOVA 600 spectrometer (operating at 599.879 MHz for ^1^H and 150.839 MHz for ^13^C) Chemical shifts are reported as δ-values in parts per million (ppm) using residual CHCl_3_ as internal reference (^1^H: δ = 7.27 ppm, ^13^C: δ = 77.23 ppm) and coupling constants (*J*) are reported in Hertz (Hz). Peak multiplicities are indicated as follows: s (singlet), d (doublet), t (triplet), q (quartet), and b (broad). Mass spectra (CI/EI/FAB) were obtained at the Department of Chemistry and Biochemistry, University of Notre Dame, using either a JEOL AX505HA or a JEOL JMS-GCmate mass spectrometer. Optical rotation data were obtained on a Perkin–Elmer polarimeter model 343 with a Na lamp.

### 3.2. 7-Hydroxy-Epothilone D Phenyl Ester (**3**)

To a solution of **2** [23] (280 mg, 0.74 mmol) in 5 mL of CH_2_Cl_2_ at −78 °C was added drop-wise 0.79 mL of TiCl_4_ (1M solution, 0.79 mmol) in CH_2_Cl_2_. The bright yellow-orange solution was stirred for 2 min, then 0.13 mL of diisopropylethylamine (DIPEA) (0.79 mmol) was added and the dark red mixture was stirred at −78 °C for 1 h. After 1 h, a solution of **1** [35] (215 mg, 0.49 mmol) in 3 mL of CH_2_Cl_2_ was added drop-wise via cannula. The reaction was left to warm to −20 °C overnight (by placing the reaction in a −78 °C bath in a freezer at −20 °C and allowing to equilibrate) and was quenched with sat. aq. NH_4_Cl. The layers were separated and the aqueous layer was further extracted with Et_2_O (3×). The organic layers were dried over MgSO_4_, filtered and concentrated in vacuo. The crude residue was purified by column chromatography using 10% EtOAc/hexanes as eluent to afford 330 mg (83%, single diastereomer) of **3**, as a clear oil. *R*_f_ = 0.56 (25% EtOAc/hexanes); ${\left[\alpha \right]}_{D}^{20}$ = −22.0 (*c* = 2.3, CHCl_3_); ^1^H NMR (600 MHz, CDCl_3_) δ 7.34–7.39 (2H, m), 7.23 (1H, m), 7.06–7.10 (2H, m), 6.92 (1H, s), 6.45 (1H, s), 5.12 (1H, dd, *J* = 7.2, 7.2), 4.50 (1H, dd, *J* = 3.6, 6.0), 4.08 (1H, dd, *J* = 6.0, 6.0), 3.46 (1H, bs), 3.32 (2H, m), 2.71 (3H, s), 2.70 (1H, dd, *J* = 4.2, 17.4), 2.56 (1H, dd, *J* = 6.0, 17.0), 2.20–2.30 (2H, m), 1.99 (3H, s), 1.96–2.02 (2H, m), 1.75 (1H, m), 1.66 (3H, s), 1.55 (1H, m), 1.45 (1H, m), 1.27 (1H, m), 1.26 (3H,s), 1.19 (3H, s), 1.07 (1H, m), 1.06 (3H, d, *J* = 6.6), 0.91 (9H, s), 0.89 (9H, s), 0.84 (3H, d, *J* = 6.6), 0.13 (3H, s), 0.11 (3H, s), 0.05 (3H, s), 0.00 (3H, s); ^13^C NMR (150 MHz, CDCl_3_) δ221.8, 170.4, 164.3, 153.2, 150.6, 142.6, 136.9, 129.4, 125.9, 121.5, 118.6, 114.9, 114.9, 79.1, 74.7, 73.3, 54.0, 41.3, 40.3, 35.5, 35.3, 32.9, 32.4, 25.9, 25.8, 25.1, 23.5, 22.5, 19.4, 19.2, 18.2, 18.2, 15.3, 13.9, 9.8, −4.2, −4.7, −4.8, −4.9; IR (NaCl, neat) 3504, 2956, 2930, 2857, 1760, 1686, 1471, 1081, 836, 777 cm^−1^; HRMS (FAB+) calculated for C_45_H_76_NO_6_SSi_2_: *m/z* = 814.4932; found 814.4942.

### 3.3. 7-O-Methylxanthate-Epothilone D Phenyl Ester (**S1**)

To a solution of **3** (150 mg, 0.18 mmol) in 2 mL of CS_2_ at −78 °C was added drop-wise 0.10 mL of NaHMDS (2.0 M in THF, 0.20 mmol). The reaction mixture was allowed to stir at −78 °C for 1.5 h, then MeI (0.11 mL, 1.8 mmol) was added drop-wise and the reaction mixture was stirred for an additional 1 h. The reaction was quenched at −78 °C with sat. aq. NH_4_Cl, diluted with CH_2_Cl_2_, and allowed to warm to ambient temperature. The layers were separated and the aqueous layer was further extracted with CH_2_Cl_2_ (2×). The combined organic layers were dried over MgSO_4_, filtered, and concentrated in vacuo. The crude residue was purified by column chromatography using 7% EtOAc/hexanes as eluent to afford 130 mg of **S1** (78%), as a slightly yellow oil. *R*_f_ = 0.50 (25% EtOAc/hexanes); ${\left[\alpha \right]}_{D}^{20}$ = −26.9 (*c* = 0.26, CHCl_3_); ^1^H NMR (600 MHz, CDCl_3_) δ 7.33–7.37 (2H, m), 7.20 (1H, m), 7.05–7.07 (2H, m), 6.92 (1H, s), 6.45 (1H, s), 5.93 (1H, dd, *J* = 4.2, 7.8), 5.13 (1H, dd, *J* = 6.6, 6.6), 4.28 (1H, dd, *J* = 4.2, 6.0), 4.07 (1H, dd, *J* = 6.0, 7.2), 3.55 (1H, dddd, *J* = 4.2, 7.2, 7.2, 13.8), 2.89 (1H, dd, *J* = 3.6, 17.4), 2.70 (3H, s), 2.56 (3H, s), 2.40 (1H, dd, *J* = 6.0, 17.4), 2.16–2.28 (2H, m), 1.99 (3H, s), 1.92–2.03 (2H, m), 1.86 (1H, m), 1.64 (3H, s), 1.44 (3H, s), 1.35–1.49 (2H, m), 1.19–1.30 (2H, m), 1.11 (3H, d, *J* = 6.6), 1.08 (3H, s), 0.97 (3H, d, *J* = 7.2), 0.91 (9H, s), 0.89 (9H, s), 0.15 (3H, s), 0.10 (3H, s), 0.04 (3H, s), 0.00 (3H, s); ^13^C NMR (150 MHz, CDCl_3_) δ 216.9, 215.1, 170.9, 164.5, 153.4, 150.8, 142.7, 136.7, 129.6, 126.0, 122.0, 121.7, 118.9, 115.2, 85.9, 79.2, 75.6, 53.6, 42.9, 40.3, 35.7, 35.6, 32.4, 32.1, 26.2, 26.1, 25.5, 23.7, 23.0, 20.9, 19.4, 18.4, 18.4, 16.3, 14.2, 11.6, −4.3, −4.4, −4.4, −4.7; IR (NaCl, neat) 2955, 2929, 2856, 1758, 1698, 1453, 1472, 1377, 1256, 1224, 1058, 836, 777 cm^−1^; HRMS (FAB+) calculated for C_47_H_78_NO_6_S_3_Si_2_: *m/z* = 904.4530; found 904.4528.

### 3.4. 7-Deoxy-Epothilone D Phenyl Ester (**4**)

A solution of **S1** (135 mg, 0.15 mmol) and 2 mg of azobisisobutyronitrile (AIBN) in 10 mL of PhMe was degassed with N_2_ for 20 min, followed by addition of 0.40 mL of HSn(*n*-Bu)_3_ (1.49 mmol). The reaction mixture was heated to 90 °C for 1 h, then allowed to cool to ambient temperature and poured onto a silica gel column. After elution of the PhMe and majority of the tin-containing byproducts with hexanes, the product was eluted using 7% EtOAc/hexanes to afford 112 mg of **4** (95%), as a colorless oil with trace HSn(*n*-Bu)_3_ impurities. *R*_f_ = 0.33 (10% EtOAc/hexanes); ${\left[\alpha \right]}_{D}^{20}$ = −9.6 (*c* = 1.0, CHCl_3_); ^1^H NMR (600 MHz, CDCl_3_, contains HSn(*n*-Bu)_3_-derived impurities) δ 7.33–7.38 (2H, m), 7.21 (1H, m), 7.06–7.10 (2H, m), 6.92 (1H, s), 6.45 (1H, s), 5.13 (1H, dd, *J* = 6.6, 7.8), 4.49 (1H, dd, *J* = 3.6, 6.6), 4.07 (1H, dd, *J* = 6.0, 6.0), 3.12 (1H, m), 2.72 (1H, dd, *J* = 3.6, 16.8), 2.71 (3H, s), 2.52 (1H, dd, *J* = 6.6, 16.8), 2.16–2.31 (2H, m), 1.99 (3H, s), 1.90–2.01 (2H, m), 1.65 (3H, s), 1.43 (1H, m), 1.25 (3H, s), 1.16 (3H, s), 1.08–1.37 (6H, m), 1.00 (3H, d, *J* = 6.6), 0.90 (9H, s), 0.89 (9H, s), 0.85 (3H, d, *J* = 6.6), 0.12 (3H, s), 0.10 (3H, s), 0.04 (3H, s), 0.00 (3H, s); ^13^C NMR (150 MHz, CDCl_3_, contains HSn(*n*-Bu)_3_-derived impurities) δ 219.0, 170.8, 164.5, 153.4, 150.9, 142.7, 137.0, 129.6, 121.7, 121.7, 118.9, 115.2, 79.2, 73.7, 53.6, 41.4, 40.5, 38.6, 37.8 *, 35.5 *, 32.4, 30.3, 28.0, 27.0, 26.2, 26.0, 25.6, 23.7, 22.6, 19.8, 19.4, 19.3, 18.4, 18.4, 17.7, 16.8 *, 14.1, 13.8 *, −4.1, −4.4, −4.5, −4.7; IR (NaCl, neat) 2929, 2856, 1760, 1698, 1493, 1471, 1252, 1197, 1144, 1084, 835, 776 cm^−1^; HRMS (FAB+) calculated for C_45_H_76_NO_5_SSi_2_: *m/z* = 798.4983; found 798.4978. * Indicates peaks derived from HSn(nBu)_3_.

### 3.5. 7-Deoxy-Epothilone D (**6**)

To a solution of **4** (100 mg, 0.13 mmol) in 0.7 mL of 4:1 THF:H_2_O was added 25 μL of H_2_O_2_ (30% in H_2_O) followed by LiOH (5 mg, 0.20 mmol). The reaction was allowed to stir at room temperature for 2 h, then carefully quenched with sat. aq. Na_2_S_2_O_3_ solution. The pH of the reaction was adjusted to 6 by the addition of 1 M HCl and the reaction was diluted with EtOAc. The layers were separated and the aqueous layer was extracted with EtOAc (3×). The organic layers were combined and dried over MgSO_4_, filtered, and concentrated in vacuo. The residue was purified by column chromatography using 25% EtOAc/hexanes containing 2% AcOH as eluent to afford 77 mg of the protected *seco* acid (85%), as a colorless oil.

To a solution of the protected *seco* acid (77 mg, 0.11 mmol) in 3.0 mL of THF at 0 °C was added 1.6 mL of HF·py (65% HF in pyridine). The reaction mixture was allowed to warm to ambient temperature over 2 h and allowed to stir for an additional 12 h. The reaction mixture was carefully poured into a solution of sat. aq. NaHCO_3_ at 0 °C and diluted with EtOAc. The layers were separated and the aqueous layer was extracted with EtOAc (4×). The combined organic layers were dried over MgSO_4_, filtered, and concentrated in vacuo. The residue was purified by column chromatography using 3% MeOH/EtOAc containing 2% AcOH as eluent to afford 44 mg of the deprotected *seco* acid (81%) as a colorless oil.

To a solution of the *seco* acid (44 mg, 0.090 mmol) in 10 mL of THF at 0 °C was added drop-wise 0.15 mL of DIPEA (0.81 mmol) followed by 43 μL of 2,4,6-trichlorobenzoyl chloride (0.27 mmol). The reaction was allowed to stir at 0 °C for 1 h, then diluted with 20 mL of THF. This mixture was added by cannula to a solution of 240 mg of 4-dimethylamino-pyridine (DMAP) (2.0 mmol) in 100 mL of PhMe over 3 h. The reaction mixture was allowed to stir at ambient temperature for 12 h, then concentrated to afford a white residue. The residue was dissolved in Et_2_O and washed successively with 20% glacial AcOH, sat. aq. NaHCO_3_, and sat. aq. NH_4_Cl. The combined organic layers were dried over MgSO_4_, filtered, and concentrated. The residue was purified by column chromatography using 25% EtOAc/hexanes as eluent to afford 35 mg of 7-deoxy-epothilone D, **6** (83%, single diastereomer) as a clear oil. R*_f_* = 0.16 (25% EtOAc/hexanes); ${\left[\alpha \right]}_{D}^{20}$ = −51.1 (*c* = 0.2, CHCl_3_); ^1^H NMR (600 MHz, CDCl_3_) δ 6.95 (1H, s), 6.58 (1H, s), 5.16 (1H, dd, *J* = 1.8, 9.0), 5.15 (1H, t, *J* = 4.8), 4.23 (1H, ddd, *J* = 3.0, 5.4, 10.2), 3.23 (1H, d, *J* = 6.0), 3.15 (1H, ddq, *J* = 4.2, 7.2, 9.0), 2.70 (3H, s), 2.64 (1H, ddd, *J* = 9.6, 9.6, 15.6), 2.44 (1H, dd, *J* = 10.2, 15.0), 2.32 (1H, dd, *J* = 3.0, 15.0), 2.27 (1H, ddd, *J* = 5.4, 9.6, 13.2), 2.20 (1H, dd, *J* = 6.6, 15.0), 2.08 (3H, d, *J* = 1.2), 1.82 (1H, ddd, *J* = 3.0, 9.6, 13.8), 1.84 (1H, m), 1.70 (3H, s), 1.65 (1H, m), 1.44 (1H, ddd, *J* = 3.6, 9.0, 12.6), 1.34 (3H, s), 1.30 (1H, m), 1.13 (1H, ddd, *J* = 4.2, 9.6, 13.8), 1.11 (3H, d, *J* = 6.6), 1.08 (3H, s), 1.02–1.08 (2H, m), 0.91 (3H, d, *J* = 6.6); ^1^H NMR (600 MHz, CD_2_Cl_2_) δ 7.00 (1H, s), 6.55 (1H, m), 5.19 (1H, dd, *J* = 7.8, 7.8), 5.13 (1H, dd, *J* = 1.2, 9.0), 4.24 (1H, ddd, *J* = 3.6, 6.6, 10.2), 3.16 (1H, ddq, *J* = 4.8, 7.2, 7.8), 3.07 (1H, bs), 2.68 (3H, s), 2.68 (1H, m), 2.39 (1H, dd, *J* = 10.2, 15.0), 2.32 (1H, m), 2.31 (1H, dd, *J* = 3.6, 15.0), 2.20 (1H, dd, *J* = 6.0, 14.4), 2.09 (3H, s), 1.87 (1H, m), 1.80 (1H, ddd, *J* = 3.0, 9.6, 13.8), 1.69 (3H, s), 1.68 (1H, m), 1.47 (1H, m), 1.35 (3H, s), 1.30 (1H, m), 1.15 (1H, ddd, *J* = 4.8, 9.6, 13.8), 1.10 (3H, d, *J* = 7.2), 1.04–1.10 (2H, m), 1.05 (3H, s), 0.93 (3H, d, *J* = 6.6); ^1^H NMR (600 MHz, DMSO-*d*_6_ δ 7.32 (1H, s), 6.48 (1H, s), 5.12–5.15 (2H, m), 5.06 (1H, bs), 4.22 (1H, d, *J* = 9.6), 3.11 (1H, ddq, *J* = 4.2, 6.6, 9.6), 2.63 (3H, s), 2.63 (1H, m), 2.24 (1H, dd, *J* = 10.8, 15.6), 2.23 (1H, m), 2.14 (1H, dd, *J* = 3.6, 15.6), 2.13 (1H, m), 2.10 (3H, s), 1.80 (1H, m), 1.63 (1H, ddd, *J* = 3.6, 9.6, 13.8), 1.61 (3H, s), 1.52 (1H, m), 1.35 (1H, m), 1.25 (1H, m), 1.17 (3H, s), 1.08 (1H, ddd, *J* = 4.2, 9.6, 13.8), 1.03–1.08 (2H, m), 1.00 (3H, d, *J* = 6.6), 0.85 (3H, s), 0.83 (3H, d, *J* = 6.6); ^13^C NMR (150 MHz, CDCl_3_) δ 218.6, 170.9, 165.1, 152.4, 139.9, 139.1, 119.9, 119.6, 116.0, 79.3, 72.8, 53.2, 41.9, 39.8, 39.4, 35.4, 32.5, 32.0, 31.2, 26.2, 23.6, 23.3, 21.2, 19.5, 19.3, 16.0; IR (NaCl, neat) 3430, 2930, 2852, 1732, 1698, 1462, 1376 cm^−1^; HRMS (FAB+) calculated for C_27_H_42_NO_4_S: *m/z* = 476.2835; found 476.2830.

### 3.6. TSE Ester Ketone Fragment (**8**)

To a solution of **S5** (100 mg, 0.224 mmol) in 5 mL of dicholoromethane (DCM) was added 2-(trimethylsilyl)ethanol (256 µL, 1.792 mmol) drop-wise followed by DMAP (164 mg, 1.344 mmol). The reaction was allowed to stir at room temperature for 36 h (reaction progress monitored by TLC), during which time the yellow color of the solution gradually faded to yield a nearly colorless solution. The reaction was quenched with sat. aq. Na_2_S_2_O_3_ solution. The layers were separated and the aqueous layer was extracted with EtOAc (3×). The organic layers were combined and dried over MgSO_4_, filtered, and concentrated in vacuo. The residue was purified by column chromatography using 5% EtOAc/hexanes as eluent to afford 80 mg of the desired ester (90%) as a colorless oil. ^1^H NMR (600 MHz, CDCl_3_) δ 4.48 (dd, *J* = 6.9, 3.8 Hz, 1H), 4.15 (ddd, *J* = 28.6, 10.9, 8.2 Hz, 2H), 2.57 (dq, *J* = 18.5, 7.2 Hz, 1H), 2.48 (dq, *J* = 18.5, 7.2 Hz, 1H), 2.41 (dd, *J* = 16.2, 3.7 Hz, 1H), 2.27 (dd, *J* = 16.2, 6.9 Hz, 1H), 1.12 (s, 3H), 1.07 (s, 3H), 1.00 (t, *J* = 7.2 Hz, 3H), 0.98 (t, *J* = 8.6 Hz, 2H), 0.84 (s, 9H), −0.06 (s, 3H), −0.04 (s, 9H), −0.02 (s, 3H); ^13^C NMR (150 MHz, CDCl_3_) δ 215.00, 172.12, 73.69, 62.81, 52.59, 39.62, 31.70, 25.86, 20.94, 20.63, 18.11, 17.20, 7.71, −1.53, −4.46, −4.96; HRMS (ESI) calculated for C_20_H_43_O_43_Si_2_: *m/z* = 403.2694; found 403.2679.

### 3.7. TSE Ester 14-(S)-Methoxy-Epothilone D (**9**)

To a solution of **8** (45 mg, 0.111 mmol) in 2.25 mL of DCM at −90 °C was added a 1 M solution of TiCl_4_ (0.013 mL, 0.119 mmol) in DCM. The solution was stirred for 5 min, then 0.020 mL of DIPEA (0.119 mmol) was added and the dark red reaction was stirred at −95 °C for 1 h. After 1 h, a solution of 45 mg of **7** (0.0742 mmol) in 1.5 mL of DCM was added drop-wise via syringe. The reaction was left to warm to −30 °C overnight (by placing the reaction bath in a freezer at −30 °C and allowing to equilibrate) and was quenched with sat. aq. NH_4_Cl. The layers were separated and the aqueous layer was extracted with Et_2_O (3×). The organic layers were combined and washed with sat. aq. NH_4_Cl. The organic layer was dried over MgSO_4_, filtered, and concentrated in vacuo. The crude residue was purified by column chromatography using 1:4 EtOAc:Hexanes as eluent to afford 35 mg (47%) of **9** as a separable mixtures of diastereomers (20:2:1). A portion of the aldehyde was recovered (10 mg, 22%, total yield 69% based on recovered starting material). ^1^H NMR (600 MHz, CDCl_3_) δ 7.64–7.59 (m, 1H), 7.38–7.27 (m, 1H), 7.27–7.21 (m, 1H), 6.76 (s, 1H), 6.38–6.36 (m, 1H), 4.99 (d, *J* = 9.4 Hz, 1H), 4.42 (dd, *J* = 6.6, 3.5 Hz, 1H), 4.29–4.24 (m, 1H), 4.20–4.10 (m, 1H), 3.96 (dt, *J* = 19.6, 9.8 Hz, 1H), 3.48 (d, *J* = 0.7 Hz, 1H), 3.27 (dd, *J* = 11.4, 5.1 Hz, 1H), 3.14 (s, 1H), 2.69 (s, 1H), 2.39 (dd, *J* = 16.5, 3.5 Hz, 1H), 2.27 (dd, *J* = 16.5, 6.6 Hz, 1H), 2.06 (ddd, *J* = 13.3, 10.5, 5.9 Hz, 1H), 1.96 (d, *J* = 1.2 Hz, 1H), 1.70 (d, *J* = 1.4 Hz, 1H), 1.61 (s, 1H), 1.54–1.36 (m, 1H), 1.35–1.25 (m, 1H), 1.24–1.20 (m, 2H), 1.19 (d, *J* = 7.8 Hz, 1H), 1.16 (dd, *J* = 4.6, 1.7 Hz, 1H), 1.13 (s, 1H), 1.03 (d, *J* = 6.9 Hz, 1H), 0.98 (dd, *J* = 8.9, 8.3 Hz, 1H), 0.87 (d, *J* = 3.0 Hz, 2H), 0.79 (d, *J* = 6.7 Hz, 1H), 0.11 (s, 1H), 0.07 (s, 1H), 0.04 (d, *J* = 3.2 Hz, 3H).

### 3.8. TSE Ester 7-O-Methylxanthate-14-(S)-Methoxy-Epothilone D (**10**)

To a solution of **9** (130 mg, 0.129 mmol) in 1.3 mL of CS_2_ (freshly distilled over CaH_2_) at −78 °C was added drop-wise 154 µL of NaHMDS solution (1.0 M in THF, 0.154 mmol). The reaction mixture was allowed to stir at −78 °C for 1.5 h, then 80 µL of MeI (1.29 mmol) was added drop-wise and the reaction mixture was stirred for an additional 1 h. The reaction was quenched at −78 °C with sat. aq. NH_4_Cl, diluted with CH_2_Cl_2_, and allowed to warm to ambient temperature. The layers were separated and the aqueous layer was extracted with CH_2_Cl_2_ (2×). The combined organic layers were dried over MgSO_4_, filtered, and concentrated in vacuo. The crude residue was purified by column chromatography using 20% EtOAc/hexanes as eluent to afford 105 mg of **10** (74%), as a slightly yellow oil. ${\left[\alpha \right]}_{D}^{20}$ = −49.5 (*c* = 0.43, CHCl_3_); ^1^H NMR (600 MHz, CDCl_3_) δ 7.61 (d, *J* = 7.9 Hz, 4H), 7.42–7.20 (m, 6H), 6.76 (s, 1H), 6.37 (s, 1H), 5.88 (dd, *J* = 8.1, 3.3 Hz, 1H), 5.00 (d, *J* = 9.4 Hz, 1H), 4.26 (d, *J* = 6.1 Hz, 1H), 4.19–4.06 (m, 3H), 3.92 (dd, *J* = 9.4, 6.1 Hz, 1H), 3.52 (dq, *J* = 6.6, 3.3 Hz, 1H), 3.13 (s, 3H), 2.69 (s, 3H), 2.56 (s, 3H), 2.09 (dd, *J* = 17.1, 6.5 Hz, 2H), 1.95 (s, 3H), 1.92–1.76 (m, 2H), 1.68 (s, 3H), 1.65 (br s, 1H), 1.42 (s, 3H), 1.41–1.36 (m, 2H), 1.27–1.25 (m, 2H), 1.21 (s, 9H), 1.08 (d, *J* = 6.6 Hz, 3H), 1.00 (s, 3H), 0.96 (dd, *J* = 8.9, 8.1 Hz, 2H), 0.91 (d, *J* = 6.8 Hz, 3H), 0.88 (s, 9H), 0.13 (s, 3H), 0.04 (s, 3H), 0.03 (d, *J* = 3.3 Hz, 9H); ^13^C NMR (150 MHz, CDCl_3_) δ 216.72, 214.68, 172.20, 163.82, 153.12, 141.10, 139.40, 135.46, 135.35, 135.29, 135.25, 129.64, 129.48, 127.33, 127.24, 124.06, 121.83, 115.23, 85.62, 80.50, 79.26, 76.15, 73.74, 62.80, 56.15, 53.22, 42.70, 39.98, 35.42, 32.66, 32.15, 31.79, 25.99, 25.93, 25.30, 23.59, 22.67, 21.18, 19.21, 18.80, 18.12, 17.21, 15.83, 15.18, 11.05, −1.53, −4.54, −4.76; HRMS (ESI) calculated for C_57_H_92_NO_8_S_3_Si_3_: *m/z* = 1098.5287; found 1098.5312.

### 3.9. Deprotected Seco Acid 7-O-Methylxanthate-14-(S)-Methoxy-Epothilone D (**12**)

To a solution of **10** (24 mg, 21.8 µmol) in 1.0 mL THF at 0 °C was added drop-wise 262 µL of TBAF solution (1.0 M in THF; 0.262 mmol) drop-wise. The reaction was allowed to stir, gradually warming from 0 °C to room temperature over 5 h. When TLC indicated complete consumption of starting material (approximately 5 h), the reaction was quenched with sat. aq. NaHCO_3_ and extracted with Et_2_O (3×). The combined organic layers were dried over MgSO_4_, filtered, and evaporated. The residue was purified by column chromatography eluting with 30% EtOAc/hexanes containing 1% AcOH to obtain 9 mg (56%) of the desired product as a white solid. The product was carried on to the next step without further characterization.

### 3.10. TBS Protected-7-O-Methylxanthate-14-(S)-Methoxy-Epothilone D (13)

To a solution of the *seco* acid (26.3 mg, 0.040 mmol) in 7.8 mL of THF at 0 °C was added drop-wise 63 µL of DIPEA (0.362 mmol) followed by 18.8 μL of 2,4,6-trichlorobenzoyl chloride (0.121 mmol). The reaction was allowed to stir at 0 °C for 1 h, then diluted with 30 mL of THF. This mixture was added by cannula to a solution of 108 mg of DMAP (0.89 mmol) in 85 mL of toluene over 3 h. The reaction mixture was allowed to stir at 40 °C for 12 h, then concentrated to afford a white residue. The residue was dissolved in Et_2_O and washed successively with 20% glacial AcOH, sat. aq. NaHCO_3_, and sat. aq. NH_4_Cl. The combined organic layers were dried over MgSO_4_, filtered, and concentrated. The residue was purified by column chromatography using 10% EtOAc/hexanes as eluent to afford 20.4 mg of **13** (78%). ${\left[\alpha \right]}_{D}^{20}$ = +30.8 (*c* = 0.89, CHCl_3_); ^1^H NMR (600 MHz, CDCl_3_) δ 7.01 (s, 1H), 6.63 (s, 1H), 6.34 (d, *J* = 10.1 Hz, 1H), 5.02 (d, *J* = 8.4 Hz, 1H), 4.91 (d, *J* = 9.2 Hz, 1H), 4.13 (dd, *J* = 8.8, 8.8 Hz, 1H), 4.03 (d, *J* = 10.2 Hz, 1H), 3.38 (dq, *J* = 10.1, 6.7 Hz, 1H), 3.23 (s, 3H), 2.77–274 (m, 1H), 2.72 (s, 3H), 2.65–2.61 (m, 1H), 2.61 (s, 3H), 2.43 (td, *J* = 11.2, 3.2 Hz, 1H), 2.18 (d, *J* = 1.2 Hz, 3H), 1.91–1.74 (m, 3H), 1.72 (d, *J* = 9.9 Hz, 3H), 1.57 (s, 1H), 1.31–1.20 (m, 1H), 1.19 (s, 3H), 1.15 (s, 3H), 1.10 (d, *J* = 6.8 Hz, 3H), 1.01 (d, *J* = 6.8 Hz, 3H), 0.97–0.89 (m, 1H), 0.85 (s, 9H), 0.11 (s, 3H), −0.12 (s, 3H); ^13^C NMR (150 MHz, CDCl_3_) δ 217.26, 212.69, 170.38, 164.65, 152.82, 142.97, 136.62, 124.51, 122.85, 116.86, 89.51, 81.85, 77.66, 76.26, 56.91, 53.63, 46.48, 39.49, 36.59, 32.78, 31.76, 27.54, 26.28, 24.56, 24.26, 23.23, 19.44, 19.32, 19.23, 18.77, 16.45, 15.60, −3.58, −5.47; HRMS (ESI) calculated for C_36_H_60_NO_6_S_3_Si: *m/z* = 726.3347; found 726.3323.

### 3.11. 7-Deoxy-14-(S)-Methoxy-TBS-Protected-Epothilone D (**14**)

A solution of **13** (13.3 mg, 0.0183 mmol) and a catalytic amount of AIBN in 1.8 mL of PhMe was degassed by bubbling N_2_ through the solution for 20 min. To the degassed solution was added 0.074 mL of HSn(*n*-Bu)_3_ (0.274 mmol). The reaction mixture was heated to 90 °C for 30 min, then allowed to cool to ambient temperature and poured onto a silica gel column. After elution of the PhMe and majority of the tin-containing byproducts using 100% hexanes, a gradient of 0% to 10% EtOAc/hexanes was used to afford 7 mg of **14** (62%) as a colorless oil containing a 91:9 ratio of the desired product and an unidentified epimer or isomer. The mixture was separated by preparative TLC eluting with 10% EtOAc/hexanes. ^1^H NMR (600 MHz, CDCl_3_) δ 6.99 (s, 1H), 6.62 (s, 1H), 5.02–4.99 (m, 2H), 4.12–4.09 (m, 2H), 3.20 (s, 3H), 3.11–3.05 (m, 1H), 2.72 (s, 3H), 2.58–2.55 (m, 2H), 2.37–2.32 (m, 1H), 2.18 (s, 3H), 1.91–1.83 (m, 2H), 1.72 (s, 3H), 1.37–1.32 (m, 1H), 1.27–1.22 (m, 1H), 1.15 (s, 3H), 1.09 (s, 3H), 1.06 (d, *J* = 6.8 Hz, 3H), 1.08–0.94 (m, 4H), 0.90 (d, *J* = 4.6 Hz, 3H), 0.84 (s, 9H), 0.06 (s, 3H), −0.12 (s, 3H). HRMS (ESI) calculated for C_34_H_58_NO_5_SSi: *m/z* = 620.3799; found 620.3797.

### 3.12. 7-Deoxy-(S)-14-Methoxy-Epothilone D (**15**)

To a solution of the protected macrolactone **14** (5.4 mg, (0.0087 mmol) in DCM (0.44 mL) at −15 °C (ice and acetone bath) was added 20% *v*/*v* TFA/DCM (87 µL) drop-wise. The mixture was allowed to warm to 0 °C and stirred for 2 h, after which time TLC indicated that all starting material had been consumed. The reaction was concentrated under reduced pressure and purified by column chromatography eluting with 50% EtOAc/Hexanes to afford 4.5 mg (100%) of the desired 7-deoxy-(*S*)-14-methoxy-epothilone D as a white solid. ${\left[\alpha \right]}_{D}^{20}$ = +6.6 (*c* = 0.06, CHCl_3_); ^1^H NMR (600 MHz, CDCl_3_) δ 6.98 (s, 1H), 6.62 (s, 1H), 5.00 (d, *J* = 9.0 Hz, 1H), 4.98 (d, *J* = 9.6 Hz, 1H), 4.20 (dd, *J* = 10.2, 3.6 Hz, 1H), 4.13 (dd, *J* = 9.6, 9.6 Hz, 1H), 3.21 (s, 3H), 3.13 (qdd, *J* = 4.8, 6.6, 9.6 Hz, 1H), 2.70 (s, 3H), 2.41 (dd, *J* = 15.0, 10.2 Hz, 1H), 2.36 (ddd, *J* = 13.8, 9.0, 4.8 Hz, 1H), 2.22 (dd, *J* = 15.0, 3.6 Hz, 1H), 2.14 (d, *J* = 1.2 Hz, 3H), 1.91 (ddd, *J* = 7.2, 7.2, 13.2 Hz, 1H), 1.79 (ddd, *J* = 13.2, 8.4, 3.0 Hz, 1H), 1.73 (d, *J* = 0.6 Hz, 3H), 1.72–1.66 (m, 1H), 1.45–1.39 (m, 1H), 1.38 – 1.33 (m, 1H), 1.32 (s, 3H), 1.27–1.25 (m, 1H), 1.19 (ddd, *J* = 13.2, 9.0, 3.0 Hz, 1H), 1.12 (d, *J* = 6.6 Hz, 3H), 1.07 (s, 3H), 0.94 (d, *J* = 7.2 Hz, 3H); ^13^C NMR (150 MHz, CDCl_3_) δ 219.07, 170.45, 165.22, 152.59, 143.96, 138.86, 124.03, 121.95, 116.54, 80.82, 78.28, 73.20, 56.67, 53.37, 41.58, 40.15, 39.41, 35.77, 32.85, 31.37, 26.47, 23.70, 23.51, 21.24, 20.89, 19.59, 19.48, 16.95; HRMS (ESI) calculated for C_28_H_44_NO_5_S: *m/z* = 506.2935; found 506.2960.

### 3.13. Cell Growth Inhibition by Compounds

Biological activity of compounds was determined by measuring inhibition of cell growth as described previously [46] using the Sulforhodamine B (SRB) method. In brief, cells that had been grown in drug-free media for 4–7 days prior to plating in 96-well plates were exposed to serial dilutions of compounds for 4 days. At the end of this period, cells were fixed, stained with SRB, retained stain quantitated by absorbance, and GI_50_ determined.

## Figures and Tables

**Table 1:** Comparative GI_50_ values of natural and synthetic microtubule stabilizers in A2780 (1A9) human ovarian carcinoma cell lines.

Compound	GI_50_ Value
Taxol^®^	0.6 nM
epothilone A	9.5 nM
epothilone B	0.2 nM
epothilone D	7.5 nM
7-deoxy-epothilone D	900 nM
7-deoxy-(*S*)-14-methoxy epothilone D	2200 nM
14-methoxy-epothilone D	29 nM
*E*-9,10-dehydro-epothilone D	<1.7 nM
zampanolide	0.25 nM

## Abbreviations

TLC	Thin layer chromatography
NMR	Nuclear magnetic resonance
AIBN	Azobisisobutyronitrile
TSE	2-Trimethylsilylethoxy
NaHMDS	Sodium bis(trimethylsilyl)amide
FDA	Food and drug administration
TBODPS	Tert-butoxydiphenylsilyl
TBAF	Tetra-*n*-butylammonium fluoride
CS_2_	Carbon disulfide
HF	Hydrogen fluoride
Bu_3_SnH	Tributyltinhydride
TBS	Tert-butyldimethylsilyl

## References

[1] Jordan M.A., Wilson L. (2004). Microtubules as a target for anticancer drugs. Nat. Rev. Cancer.

[2] Jordan M.A., Wilson L. (1990). Kinetic analysis of tubulin exchange at microtubule ends of low vinblastine concentrations. Biochemistry.

[3] Hastie S.B. (1991). Interactions of colchicine with tubulin. Pharmacol. Ther..

[4] Fanale D., Bronte G., Passiglia F., Calo V., Castiglia M., di Piazza F., Barraco N., Cangemi A., Catarella M.T., Insalaco L. (2015). Stabilizing versus destabilizing the microtubules: A double-edge sword for an effective cancer treatment option?. Anal. Cell. Pathol..

[5] Komlodi-Pasztor E., Sackett D., Wikerson J., Fojo T. (2011). Mitosis is not a key target of microtubule agents in patient tumors. Nat. Rev. Clin. Oncol..

[6] TAXOL^®^ (Paclitaxel) for Injection. www.rxlist.com/taxol-drug.html.

[7] Höfle G., Bedorf N., Steinmetz H., Schomburg D., Gerth K., Reinchenbach H. (1996). Epothilone A and B-Novel 16-membered macrolides with cytotoxic activity: Isolation, crystal structure, and conformation in solution. Angew. Chem. Int. Ed..

[8] Gerth K., Bedorf N., Höfle G., Irschik H., Reinchenbach H. (1996). Antibiotics from gliding bacteria. 74. Epothilons A and B: Antifungal and cytotoxic compounds from *Sorangium cellulosum* (myxobacteria): Production, physic-chemical and biological properties. J. Antibiot..

[9] Bollag D.M., McQueney P.A., Zhu J., Hensens O., Koupal L., Liesch J., Goetz M., Lazarides E. (1995). Woods, Epothilones, a new class of microtubule-stabilizing agents with a taxol-like mechanism of action. Cancer Res..

[10] Forli S., Manetti F., Altmann K.H., Botta M. (2010). Evaluation of novel epothilone analogues bymeans of a common pharmacophore and a QSAR pseudoreceptor model for taxanes and epothilones. Chem. Med. Chem..

[11] Kumar A., Heise H., Blommers M.J., Krastel P., Schmitt E., Petersen F., Jeganathan S., Mandelkow E.M., Carlomagno T., Griesinger C. (2010). Interaction of epothilone B (patupilone) with microtubules as detected by two-dimensional solid-state NMR spectroscopy. Angew. Chem. Int. Ed..

[12] Altmann K., Gaugaz F.Z., Schiess R. (2011). Diversity through semisynthesis: The chemistry and biological activity of semisynthetic epothilone derivatives. Mol. Divers..

[13] Nicolaou K.C., Ritzen A., Namoto K. (2001). Recent developments in the chemistry, biology and medicine of the epothilones. Chem. Commun..

[14] IXEMPRA® Kit (Ixabepilone) for Injection [Package Insert]. www.ixempra.com.

[15] Nicolaou K.C., Vourloumis D., Li T., Pastor J., Winssinger N., He Y., Ninkovic S., Sarabia F., Vallberg H., Roschangar F. (1997). Designed epothilones: Combinatorial synthesis, tubulin assembly properties, and cytotoxic action against taxol-resistant tumor cells. Angew. Chem. Int. Ed..

[16] Su D., Balog A., Meng D., Bertinato P., Danishefsky S.J., Zheng Y., Chou T., He L., Horwitz S.B. (1997). Structure-activity relationship of the epothilones and the first in vivo comparison with paclitaxel. Angew. Chem. Int. Ed..

[17] Nicolaou K.C., Roschangar F., Vourloumis D. (1998). Chemical biology of epothilone. Angew. Chem. Int. Ed..

[18] Larsen E.M., Wilson M.R., Taylor R.E. (2015). Conformation-activity relationships of polyketide natural products. Nat. Prod. Rep..

[19] Taylor R.E., Zajicek J. (1999). Conformational properties of epothilone. J. Org. Chem..

[20] Yoshimura F., Rivkin A., Gabarda A.E., Chou T.-C., Dong H., Sukenick G., Morel F.F., Taylor R.E., Danishefsky S.J. (2003). Synthesis and Conformational Analysis of (*E*)-9,10-Dehydro Epothilone B. A Suggestive Linkage between Observations in the Chemistry and Biology of Epothilones. Angew. Chem. Int. Ed..

[21] Taylor R.E., Chen Y., Beatty A., Myles D.C., Zhou Y. (2003). Conformational-activity relationships in polyketide natural products: A new perspective on the rational design of epothilone analogues. J. Am. Chem. Soc..

[22] Taylor R.E., Chen Y., Galvin G.M., Pabba P.K. (2004). Conformation-activity relationships in polyketide natural products. Towards the biologically active conformation of epothilone. Org. Biomol. Chem..

[23] Frein J.D., Taylor R.E., Sackett D.L. (2009). New sources of chemical diversity inspired by biosynthesis: Rational design of a potent epothilone analogue. Org. Lett..

[24] Giannakakou P., Gussio R., Nogales E., Downing K.H., Zaharevitz D., Bollbuck B., Poy G., Sachett D., Nicolaou K.C. (2000). A common pharmacophore for epothilone and taxanes: Molecular basis for drug resistance conferred by tubulin mutations in human cancer cells. Proc. Natl. Acad. Sci. USA.

[25] Manetti F., Forli S., Maccari L., Corelli F., Botta M. (2003). 3D QSAR studies of the interaction between β-tubulin and microtubule stabilizing antimitotic agents (MSAA). A combined pharmacophore generation and pseudoreceptor modeling approach applied to taxanes and epothilones. Farmaco.

[26] Manetti F., Maccari L., Corelli F., Botta M. (2004). 3D QSAR models of interactions between β-tubulin and microtubule stabilizing antimitotic agents (MSAA): A survey on taxanes and epothilones. Curr. Top. Med. Chem..

[27] Carlomagno T., Sánchez V.M., Blommers M.J.J., Griesinger C. (2003). Derivation of dihedral angles from CH–CH dipolar-dipolar cross-correlated relaxation rates: A C–C torsion involving a quaternary carbon atom in epothilone A bound to tubulin. Angew. Chem. Int. Ed..

[28] Carlomagno T., Blommers M.J.J., Meiler J., Jahnke W., Schupp T., Petersen F., Schinzer D., Altmann K., Griesinger C. (2003). The high-resolution solution structure of epothilone A bound to tubulin: An understanding of the structure-activity relationships for a powerful class of antitumor agents. Angew. Chem. Int. Ed..

[29] Lange A., Schupp T., Petersen F., Carlomagno T., Baldus M. (2007). High-resolution solid-state NMR structure of an anticancer agent. ChemMedChem.

[30] Erdélyi M, Pfeiffer B., Hauenstein K., Fohrer J., Gertsch J., Altmann K., Carlomagno T. (2008). Conformational preferences of natural and C3-modified epothilones in aqueous solution. J. Med. Chem..

[31] Sefkow M., Kiffe M., Schummer D., Hofle G. (1998). Oxidative and reductive transformations of epothilone A. Bioorg. Med. Chem. Lett..

[32] Nicolaou K.C., Ninkovic S., Sarabia F., Vourloumis D., He Y., Vallberg H., Finlay M.R.V., Yang Z. (1997). Total syntheses of epothilone A and B via a macrolactonization-based strategy. J. Am. Chem. Soc..

[33] Schinzer D., Bauer A., Schieber J. (1999). Synthesis of (−)-epothilone B. Chem. Eur. J..

[34] Mulzer J., Mantoulidis A., Ohler E. (2000). Total syntheses of epothilones B and D. J. Org. Chem..

[35] Taylor R.E., Chen Y. (2001). Total synthesis of epothilones B and D. Org. Lett..

[36] Koch G., Loiseleur O., Fuentes D., Jantsch A., Altmann K.-H. (2002). Diastereoselective titanium enolate aldol reaction for the total synthesis of epothilones. Org. Lett..

[37] Barton D.H.R., McCombie S.W. (1975). A new method for the deoxygenation of secondary alcohols. J. Chem. Soc. Perkin Trans. I.

[38] Inanaga J., Hirata K., Saeki H., Katsuki T., Yamaguchi M. (1979). A rapid esterification by means of mixed anhydride and its application to large-ring lactonization. Bull. Chem. Soc. Jpn..

[39] Hardt I.H., Steinmetz H., Gerth K., Sasse F., Reichenbach H., Höfle G. (2001). New natural epothilones from *Sorangium cellulosum*, strains So ce90/B2 and So ce90/D13: Isolation, structure elucidation, and SAR studies. J. Nat. Prod..

[40] Prota A.E., Bargsten K., Zurwerra D., Field J.J., Diaz J.F., Altmann K.H., Steinmetz M.O. (2013). Molecular mechanism of action of microtubule-stabilizing anticancer agents. Science.

[41] Canales A., Nieto L., Rodríguez-Salarichs J., Sánchez-Murcia P.A., Coderich C., Cortés-Cabrera A., Patterson I., Carlomagno T., Gago F., Andreu J.M. (2014). Molecular recognition of epothilones by microtubules and tubulin dimers revealed by biochemical and NMR approaches. ACS Chem. Biol..

[42] Ding F., Jennings M.P. (2008). Total synthesis of (−)-dactylolide and formal synthesis of (−)-zampanolide via target oriented β-*C*-glycoside formation. J. Org. Chem..

[43] Wilson M.R. (2014). Synthesis, Conformational Analysis, and Biological Evaluation of the Potent Microtubule-Stabilizing Agents (−)-Zampanolide and (−)-Dactylolide. Ph.D. Thesis.

[44] Daly E.M. (2011). Probing Tubulin Interactions with 14-Methyl Epothilone D Analogues. Ph.D. Thesis.

[45] Bissantz C., Kuhn B., Stahl M. (2010). A Medicinal Chemist’s Guide to Molecular Interactions. J. Med. Chem..

[46] Begaye A., Trostel S., Zhao Z., Taylor R.E., Schriemer D.C., Sackett D.L. (2011). Mutations in the β-tubulin binding site for peloruside A confer resistance by targeting a cleft significant in side chain binding. Cell Cycle.

